# Social Stigma and Depression among Asymptomatic COVID-19 Carriers in Shanghai, China: The Mediating Role of Entrapment and Decadence

**DOI:** 10.3390/ijerph192013006

**Published:** 2022-10-11

**Authors:** Hui Chen, Yingjie Chen, Yinghuan Zhang, Zhiqiang Wang, Dake Shi, Jialin Liu, Xiaodong Yang, Lulu Xu, Yong Cai, Fan Hu

**Affiliations:** 1 School of Public Health, Shanghai Jiao Tong University School of Medicine, Shanghai 200025, China; 2 Department of Infection Control, Ruijin Hospital, Shanghai Jiao Tong University School of Medicine, Shanghai 200025, China; 3 Department of Critical Care Medicine, Ruijin Hospital, Shanghai Jiao Tong University School of Medicine, Shanghai 201801, China; 4 Department of Neurology and Institute of Neurology, Ruijin Hospital, Shanghai Jiao Tong University School of Medicine, Shanghai 200025, China

**Keywords:** asymptomatic COVID-19 carriers, social stigma, depression, entrapment, decadence, mediating effect

## Abstract

Introduction: Since the advent of 2019 novel coronavirus (COVID-19), the coexistence between social stigma and depression symptoms (depression hereafter) in COVID-19 patients has been mentioned, but the mechanisms involved remains unclear. This study aimed to explore how the stigma affects depression during the mid-pandemic period. Methods: A cross-sectional survey using non-probability sampling was conducted among asymptomatic COVID-19 carriers in Shanghai, China (April 2022). An online questionnaire was used to obtain information on demographic characteristics and psychological traits. Logistic regression and path analysis were performed to analyze the depression risk factors and examine the mediation model, respectively. Results: A total of 1283 participants (59.6% men) were involved in this study, in which 44.7% of carriers reported having depression. Univariate analyses found that education level (OR 0.575; 95% CI 0.448–0.737) and doses of vaccine (OR 1.693; 95% CI 1.042–2.750), were significantly associated with depression among asymptomatic carriers. The association between social stigma and depression was fully mediated by their feelings of entrapment and decadence (indirect effect = 0.204, *p* < 0.001; direct effect = −0.059, *p* = 0.058). The mediating role of entrapment between stigma and depression was moderated by age group (estimate = 0.116, *p* = 0.008). Conclusion: Mental health issues resulting from the COVID-19 pandemic are increasingly apparent in China and require urgent attention and responses. These findings provide new perspectives for the early prevention of depression in asymptomatic carriers.

## 1. Introduction

The pandemic of 2019 novel coronavirus (COVID-19) has become a major global public health challenge [1,2]. It is self-evident that it not only caused the burden of physical illness but has also triggered a wide range of declines in mental well-being [3,4]. A meta-analysis suggested that the global prevalence of major depressive disorder increased the most among a series of negative psychology issues with the emergence of COVID-19. The prevalence of depression increased by 27.6% (25.1% to 30.3%), with a loss of disability-adjusted life-years of 4.94 million (33.6 to 68.7) [5]. Global levels of depression in the general population, health care providers and patients during the COVID-19 pandemic went up to 24.0% (21.0–27.1%) [6]. A meta-analysis revealed that psychological problems in infected patients were prominent, with a prevalence of 45% depression [7].

Although risk factors for depression in vulnerable groups have been documented [8,9,10], current information regarding how the factors affect depression is lacking. Evidence suggested that COVID-19 survivors experienced higher stigma levels compared with healthy controls [11]. COVID-19-related stigma refers to a disagreeable or negative self-attitude that develops after infection or close contact with COVID-19 patients [12], including the negative deflection of social identity, with self-concept thus leading to a “spoiled identity” [12,13]. Additional evidence suggests a correlation between depression and social stigma. Liu et al. emphasized the importance of stigma in exacerbating the emotional impact of infected patients [9]. A prospective cohort study of COVID-19 survivors found that discrimination may partly account for the high incidence of depression after infection remission [14]. The identity-threat model of stigma raised by Brenda Major et al., which views stigma as a stressor, attempts to explain the effects of stigma on psychological well-being such as depression through coping strategies [15,16].

The economic consequences and psychosocial impacts of the COVID-19 pandemic are pervasive and profound [17,18,19]. Consequently, to avoid long-term mental health issues, there is an urgent need to clarify the mechanisms of the development of depression for COVID-19 patients [20,21]. The Social Rank Theory (SRT) proposes that depression evolves as a natural result of prolonged involuntary subordination [22]. Faced with an unfavorable situation (e.g., unachievable objectives, attempted change or challenge inhibited), individuals involuntarily yield adaptive responses through an automatic shutdown strategy [23]. If the situations persist and cannot be changed or escaped, the adaptive response will progressively change to maladjustment and eventually lead to depression [24,25]. Entrapment and decadence are precisely the key maladaptive defensive responses to this process. Entrapment is described as a common situation when there is a strong motivation to escape from an unsatisfying status, but that expectation cannot be met [22,26]. It could activate the Involuntary Defeat Strategy that, along with the feelings of decadence, forms a “depressogenic feedback loop” and ultimately contributes to the development of depression [27].

To our knowledge, little is known about the exact mechanisms on how stigma affects depression. Aiming to address this gap in understanding, we hypothesized and constructed a mediating model in which entrapment and decadence are mediators between social stigma and depression. The reasons are as follows: First, the identity-threat model of stigma posits that intrusive thinking and rumination about an event or issues occur when stigmatized individuals respond to stigma with involuntary engagement [15,28]. These two responses may have potential connection points with the assumed prerequisites of the SRT, that adverse situations are uncomfortable for individuals but cannot yet be accepted or escaped. That is, the identity-threat model of stigma implies that stigma affects entrapment and decadence. Second, entrapment and decadence are psychological signals prior to the onset of depression based on the SRT. Finally, a stigma-related stressor begins with external stimuli, while entrapment or decadence are inherently intrinsic psychological products, which satisfies the theoretical requirement of chronological order.

The primary objective of this study was to explore potential mediations between social stigma and depression via entrapment or decadence based on quantitative data from asymptomatic COVID-19 carriers in Shanghai, China. These findings may help us further understand the impact of the pandemic on COVID-19 patients’ mental health and inform subsequent intervention strategies and services for depression related to COVID-19.

## 2. Methods 

### 2.1. Study Design and Participants

This is a cross-sectional study of asymptomatic COVID-19 carriers admitted to the Ruijin Jiahe Fangcang shelter hospital (Shanghai, China) in April 2022. Non-probability sampling was used to recruit participants for this study. Health care providers were responsible for advocacy and inviting asymptomatic patients, aiming to cover over 80% of patients admitted. According to previous studies, the expected prevalence of depression among COVID-19 carriers was 45% [7]. We used α of 0.05 and a permissible error of 0.03. Considering a non-response rate of 10%, the required sample size was 1209.

Inclusion criteria included: (1) older than 18 years. (2) A diagnosis of asymptomatic COVID-19 infection, which was judged by the threshold Cycle (Ct) value obtained from the real-time reverse transcriptase-polymerase chain reaction (RT-PCR) test. The test for SARS-CoV-2 RNA in the nasopharynx is considered positive at a CT value below 35. (3) Able to use WeChat to complete the questionnaire independently. (4) Capable of providing informed consent. The study was approved by the Ethics Committee of Ruijin Hospital, Shanghai Jiao Tong University School of Medicine (protocol code LL202070).

### 2.2. Data Collection 

To comply with social distancing guidelines, an electronic questionnaire was selected for assessment in this study. Health care providers asked participants to answer questions based on their feelings and thoughts in the recent period when the diagnosis of COVID-19 was confirmed and presented QR codes of the questionnaire to the participants. Self-designed questionnaires were administered to collect information about demographic characteristics and psychological traits (social stigma, entrapment, decadence and depression). The information was gathered via the online survey platform “Questionnaire Star”. All survey respondents provided their informed written consent before the survey.

### 2.3. Measures

#### 2.3.1. Background Characteristics 

Information on background characteristics was collected, including age, sex, education level, marriage status, length of time from diagnosis, and doses of COVID-19 vaccines.

#### 2.3.2. Social Stigma

Stigma from COVID-19 was measured via two subscales of the Social Impact Scale (SIS) [29], a generic stigma scale initially applied to patients with cancer or infectious diseases (e.g., human immunodeficiency virus, HIV) (Cronbach’s α = 0.85–0.90). Sixteen items assessed the social stigma related to COVID-19 at the mid-stage of the outbreak. (1) Social rejection measures stigma-related stressors, including traditional infectious disease’s role beliefs (perceiving others to treat them with less respect, no longer consider them competent or avoid them), and experiencing a major life event (being denied employment, education or being otherwise neglected) [16,29]. It gives the individual a sense of being discriminated against at work and in society [29]. (2) Social isolation signifies a sense of anomie in the traditional sociological sense, including feelings of loneliness, inequality with others and uselessness, accompanied by a devalued social identity [16,29]. Compared to HIV stigma, the stigmatization of COVID-19 has less of a moral link and perhaps less self-blame than HIV [30]. In addition, the trajectory of people diagnosed as positive needs to be disclosed in order to reduce public panic. We therefore believe that internalized shame from the SIS is not sufficiently consistent with the content of the social stigma of individuals at the early stage of diagnosis. Thus, only two subscales of the SIS were included. Sample items included “my employer/co-workers have discriminated against me because of my illness”, “some people act as though I am less competent than usual.” The options were rated from 1 (strongly disagree) to 4 (strongly agree). Higher total scores suggested higher levels of COVID-19-related stigma. A score lower than the 75th percentile—a score of 35—was defined as a low level. The Chinese version of the SIS has been found to have good psychological traits [31]. The SIS was applied to measure COVID-19-related stigma in Chinese COVID-19 survivors in 2020 [11]. The Cronbach’s α of the SIS in this study was 0.967, with factor loadings of 0.511–0.745. Psychometrics for the current study sample are enclosed in the Supplementary Materials.

#### 2.3.3. Entrapment and Decadence

Feelings of entrapment was assessed by the Chinese version of Entrapment Scale (ES) [32] (Cronbach’s α = 0.96). The ES is a 16-item self-report scale used to identify the subjective experiences of entrapment [22]., e.g., “I am in a situation I feel trapped in”. Options for each item range from 0 (not at all), 1 (light), 2 (medium), 3 (heavy), and 4 (serious) [33]. The total possible score can be between 0 and 64. A level above the 75th percentile was defined as a high level of sense of entrapment.

Feelings of decadence were quantified with the Chinese version of the subscale of Defeat Scale (DS) (Cronbach’s α = 0.93), which is designed to assess personal perceptions of failed struggles and loss of rank within the last week [22]., e.g., “I feel defeated by life”. The response options are 0 (never), 1 (seldom), 2 (sometimes), 3 (often), and 4 (always). The overall scores for this 13-item scale range from 0 to 52. A total score over the 75th percentile was defined as a high level of decadence. Both scales have demonstrated validity and reliability among Chinese populations [32,34]. In the present study, the internal consistency coefficients of ES and DS were 0.973 and 0.904, respectively, and the factor loadings of each item in confirmatory factor analysis were roughly above 0.7. Details are presented in the Supplementary Materials.

#### 2.3.4. Depression

The Zung Self-Rating Depression Scale (SDS), which consists of 20 self-report items, was used to assess participants’ depression based on their feelings [35]. The SDS scale includes physiological and psychological symptoms which have been identified in factor analysis studies of depression [35]. Ten items reflect the negative aspect., e.g., “I feel down-hearted and blue”. Ten items express positive aspects and are reverse scored., e.g., “morning is when I feel the best”. Each question has four response options, from 1 (none, or a little of the time) to 4 (most, or all of the time). The final raw score is the sum of the 20 items, ranging from 20 to 80. A higher score indicates a higher level of depressive severity. The total raw score ≥ 42 is taken as presenting with depression. Additionally, the cut-off value has also been adopted in other Chinese studies [36,37]. The Chinese version of the SDS has been shown to be a valid tool for screening depression (Cronbach’s α = 0.82) [36,38].

### 2.4. Statistical Analysis

The normally distributed variable (age) and skewedly distributed variables (stigma, entrapment, decadence, and depression) were described as the mean with standard deviation (SD) and median (interquartile range (IQR)), respectively. Categorical variables (e.g., sex and education level) were expressed as counts and percentages. The univariate logistic regression model was used to identify the characteristics associated with depression.

The Change-in-Estimate procedure was used for confounder identification and selection [39]. All socio-demographic variables and the target variable (stigma) were included in a multivariate logistic regression to build the initial full model. Covariates were selected by backward elimination. In this procedure, the covariate for which removal caused a change of less than 10% in the OR of stigma was removed [39]. The correlation coefficients between variables in final model were based on Spearman correlation analysis. We performed three hierarchical regression analyses to initially test the mediating effect. In step 1, the association of significant sociodemographic characteristics to depression was tested. The stigma factor was added in the step 2. Entrapment; decadence; and entrapment and decadence were added separately in step 3 to test for changes in the effect of stigma on depression.

After adjusting for covariates, path analysis was conducted to examine the relationships among stigma, entrapment, decadence, and depression. Four psychosocial variables are expressed as quantitative data in the mediation model. Two product-of-coefficients strategies (bias-corrected and percentile methods) were performed to examine the mediating role of entrapment and decadence in the association between stigma and depression [40]. In this process, 5000 bootstrap samples were used. The 95% confidence intervals (CI) that do not contain zero indicate the significance of the indirect effects [40,41]. Finally, a bias-corrected bootstrapping procedure based on 5000 resamples was employed to examine the significance of the age-moderated mediation effect. Using the mean value as the cut-off point, age was divided into high and low groups in the moderation analysis. The high age group mentioned in this study is only relative to the low group.

To enhance the credibility of our analysis, a temporal validation was conducted [42]. The data were randomized to the training group and validation group in a 3:1 ratio. Then, the bootstrapping method was used for temporal validation.

Descriptive analyses, logistic regression, correlation analysis and hierarchical regression were conducted using IBM SPSS Statistics 26.0 (IBM Corp., Armonk, NY, USA). Path analysis was conducted using R software (V. 4.2.1; http://www.Rproject.org; URL (accessed on 1 June 2022)). *p* values < 0.05 were considered as statistically significant.

## 3. Results

### 3.1. Participants

**Figure 1:**
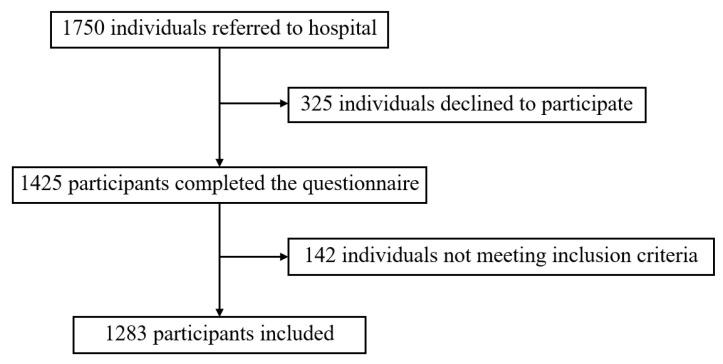
Participants’ recruitment flow chart.

Of 1750 individuals referred to hospital, 1425 completed the questionnaire. The response rate was 81.4%. After excluding 142 participants who did not meet the inclusion criteria, a total of 1283 participants were included, as shown in Figure 1.

### 3.2. Sociodemographic Characteristics

Table 1 shows the demographic and psychosocial characteristics of 1283 asymptomatic COVID-19 carriers during the COVID-19 outbreak in Shanghai. The mean age of participants was 39.6 ± 11.1 years with a range from 18 to 71, and 765 (59.6%) were men. The majority of participants were married (948 (73.9%)) and graduated from senior secondary or below (906 [70.6%]). More than eighty percent of participants (1097) were diagnosed as asymptomatic carriers for greater than or equal to 8 days, among which, 646 (50.4%) tested positive for SARS-CoV-2 RNA within two weeks. Regarding COVID-19 vaccination, a large number of participants received the vaccine, of which, 808 (63.0%) received three doses, 355 (27.7%) received two doses, and less than ten percent of participants (9.4%) did not receive the vaccine or only received a single dose.

In the univariate analysis (Table 1), education level (OR 0.575; 95% CI 0.448–0.737) and doses of vaccine (OR 1.693; 95% CI 1.042–2.750) were significantly associated with depression among asymptomatic COVID-19 carriers. Higher educational attainment was a protective factor, whereas three doses of vaccine was a risk factor for depression.

### 3.3. Covariate Selection and Correlation Analysis

Considering the significant effect of education level and vaccination dose on depression in both univariate and multivariate logistic regressions, these two covariates were included in the subsequent analysis (Supplementary Materials Table S7). The Change-in-Estimate procedure showed that by sequentially removing the socio-demographic variables (the four remaining variables), none of the ORs of the stigma changed by more than 10%; thus, all met the criteria for exclusion (Supplementary Materials Table S8).

As shown in Table 2, the median perceived stigmatization score for all participants was 29.0 (IQR = 18.0), with 305 participants (23.8%) reaching a high level. Participants’ median entrapment and decadence scores were 1.0 (IQR = 10.0) and 2.0 (IQR = 12.0), among which, 318 (24.8%) and 304 (23.7%) reported high levels, respectively. With respect to depression, the median score of the SDS was 40.0 (IQR = 19.0), and nearly half of the participants (574 (44.7%)) reported having depression.

Education level was significantly associated with all other variables. Doses of vaccine was significantly associated with stigma. Stigma was positively correlated with depression, entrapment and decadence. Depression was positively correlated with entrapment and decadence, and entrapment was positively correlated with decadence.

### 3.4. Hierarchical Regression Analysis

Our data met the assumption for mediation. Significant sociodemographic characteristics, including education level and doses of vaccine tested in step 1, explained 2.0% of the variance in depression. Stigma scores, added in step 2, had significant effects on depression (β = 0.148, t = 5.551, *p* < 0.001). However, the effect was no longer significant after the addition of entrapment and decadence in step 3 (*p* > 0.05) (Table 3).

### 3.5. Path Analysis

Adjusted for covariates (i.e., education level, doses of vaccine), the results of the path analysis are detailed in Table 4. The indirect path from perceived stigma to depression through entrapment *(p* < 0.001) and decadence (*p* = 0.001) were significant, while the direct path between them was not significant (*p* = 0.058). In another word, entrapment and decadence completely mediated the relationship between stigma and depression (estimate = 0.204, Delta z = 10.912, *p* < 0.001).

**Figure 2:**
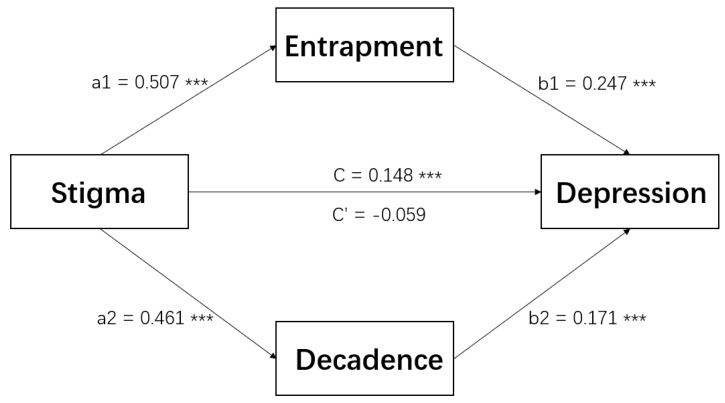
The mediating role of entrapment and decadence in the relationship between stigma and depression (*n* = 1283). *** *p* < 0.001. Note: education level and doses of vaccine significantly associated with depression in logistic regression were controlled as covariates in the path analysis. C’: change in coefficient when mediating variables are incorporated.

As shown in Figure 2, stigma among COVID-19 carriers was positively associated with their feelings of entrapment (estimate = 0.507, *p <* 0.001), which in turn was positively associated with their risk of depression (estimate = 0.247, *p* < 0.001). Similarly, the social stigmatization of participants was significantly linked to the sense of decadence (estimate = 0.461, *p* < 0.001), which in turn was positively related to depressive symptoms (estimate = 0.171, *p* < 0.001). When comparing indirect effects in the mediator models of entrapment and decadence, the differences were not significant (*p* = 0.254) (Table 3).

The mediating role of entrapment between stigma and depression was moderated by age group (estimate = 0.116, *p* = 0.008). For participants aged 18–39 years, the indirect effect of entrapment was significant (estimate = 0.182, Delta z = 4.962, *p* < 0.001). For subjects aged 40–71 years, the mediating role of entrapment was still positively correlated with stigma and depression, but much weaker (estimate = 0.066, Delta z = 2.754, *p* = 0.006). However, the moderating role of age group in the decadence path was not significant (estimate = −0.114, *p* = 0.762) (Table 3).

### 3.6. Temporal Validation

We additionally considered the relationship between one variable and all other variables in the model in the training set; the path plot is shown in Supplementary Materials Figure S1. The results of the validation set showed that entrapment and decadence partially mediated the relationship between stigma and depression (Supplementary Materials Table S9).

## 4. Discussion

We conducted a quantitative study to explore the potential mechanism of how stigma affects the likelihood of depression in asymptomatic carriers during the mid-stage of the COVID-19 outbreak in Shanghai. In the current study, depression was reported by 44.7% of participants. A high education level was a protective factor for depression. This study characterized the psychological status of COVID-19 asymptomatic carriers, underscoring the importance of psychiatric screening and early interventions in this subgroup. Moreover, we found a mediating role of entrapment and decadence on the relationship between social stigma and depression, which offers a new way to mitigate the high prevalence of depression among asymptomatic carriers in China.

We found that the prevalence of depression reported by asymptomatic carriers was comparable to the rate in COVID-19 patients in previous studies [7,43]. Since physicians prioritize the physical illnesses of hospital patients, it is not surprising that the mental health issues of this subpopulation did not receive attention. Asymptomatic carriers account for a large proportion of COVID-19 patients in China, which highlights the importance of raising awareness of psychiatric screening in infected patients. In addition, the final model showed that a higher education level was a protective factor for depression. Inconsistent findings were reported for the association between education level and psychological symptoms [7,9]. One explanation for our finding is that better-educated individuals tend to acquire the correct information and awareness about COVID-19, therefore being more likely to avoid some adverse psychological health outcomes.

In line with our hypothesis, entrapment and decadence mediate the relationship between stigma and depression in asymptomatic carriers during the epidemic. People who suffered from stigma were more likely to feel trapped and decadent, which, in turn, was associated with an increase depressive symptoms. Two conceptual frameworks described at the beginning of the article guided this mediation assumption complementarily. Moreover, we found that the indirect relationship between stigma and depression through entrapment was moderated by age group. This further supports the finding that stigma can affect depression via entrapment.

Mediation analysis is essentially a correlation analysis [44]. Based on accumulated theory and research, we hypothesized that entrapment mediates the relationship between stigma and depression. However, the existence of a correlation between stigma and depression may also be the result of reverse causality or the presence of confounding factors. We found a significant difference in the correlation coefficients between stigma and depression in the two age groups, which at least suggests that the correlation is not exactly the result of the two possible causes mentioned above (reverse causality and confounding factors) [44,45]. Otherwise, their correlation would not differ. Thus, the mediation effect exists.

Our findings have three implications for the early detection of depression and effective mental health services for asymptomatic carriers. First and foremost, the timely assessment of stigmatizing stressors associated with COVID-19 must be carried out. Prior studies suggest that social stigma is not only a product of structural inequalities caused by bias and discrimination, but also the result of individual failures to cope adaptively with the stressors [15]. Ding K et al. also suggested that personal-level factors are more likely to be related to depression [46,47]. Therefore, the consideration of individuals’ stigmatizing stressors in early depression interventions is clearly warranted. This study focused on stigmatizing stressors related to job refusals and other overlooked situations. In future research, we believe there is a need to help infected individuals identify the well-defined type of stigma-related stress (e.g., being quarantined or treatment factors) they encounter to effectively help them cope with it successfully.

Second, response efforts to alleviate entrapment and decadence can be a crucial early intervention point for depression. Our main results shows that entrapment and decadence fully mediated the association between COVID-19-related stigma and depression. It appears that improving entrapment and decadence may effectively block the effects of stigma on depression. Specifically, an assessment of the psychological status of entrapment and decadence can be included in the early detection of depressive symptoms. Additionally, hospitals can implement remote mental health psychiatric counseling and screening programs through telemedicine or online mental health interventions [48,49]. Preventive strategies and early interventions are needed to adequately address early psychological abnormal states and avoid long-term mental health problems.

Third, this research provides a basis for implementing individualized mental health intervention strategies. Our findings revealed that the indirect link between stigmatization and depression via entrapment is stronger in younger age groups, which implies we could give priority to younger infected individuals with respect to entrapment-reducing interventions. Meanwhile, we also need to find other influential factors of the mental state of entrapment and decadence for relatively high age groups in the future.

The current study involves several limitations. First, participants were recruited by non-probability sampling approaches, which may be subject to sampling bias. The non-response rate in this study was 18.4%, which may underestimate the prevalence of depression. Second, participants were recruited from one location, and we need to be cautious when applying the results to other areas. However, we did use a larger sample in our study, so the results may provide a worthy reference for the exploration of depressive mechanisms. Third, the cross-sectional study design limits our ability to establish causal mediation relationships. The mediating effect was developed and temporally validated based on the observational data. Future studies should trace changes in the psychological status of carriers at 376 later stages to further verify the causal mechanism. Fourth, other stigma-related stressors such as isolation factors or treatment factors were not studied. Future research could consider more stressors into account to understand the mechanism in depth.

## 5. Conclusions

This is one of the few studies to provide insights into the relationship between social stigma and depression among asymptomatic COVID-19 carriers in the post-epidemic era. Asymptomatic carriers were more likely to exhibit depression in the mid-stage of the pandemic. The current data showed the linkage between social stigma and depression through the mediating effects of entrapment and decadence. The routine assessment of stigma and screening for entrapment and decadence should be incorporated into early intervention strategies for depression among carriers to alleviate the psychological burden of the outbreak on society.

## Figures and Tables

**Table 1:** Characteristics of asymptomatic COVID-19 carriers and univariate logistic regression analysis of depression (*n* = 1283).

Variables	Participants N (%)	Depression	
		OR (95% CI)	*p* Value
Age (years) mean ± SD	39.6 ± 11.1	1.003 (0.993–1.013)	0.535
Sex			
Men	765 (59.6)	Reference	-
Women	518 (40.4)	0.940 (0.751–1.176)	0.587
Education level			
Senior secondary or below	906 (70.6)	Reference	
College or above	377 (29.4)	0.575 (0.448–0.737)	<0.001
Marriage status			
Unmarried	285 (22.2)	Reference	-
Married	948 (73.9)	1.060 (0.811–1.384)	0.671
Divorced or widowed	50 (3.9)	1.199 (0.656–2.189)	0.556
The length of time from diagnosis			
≤7 days	186 (14.5)	Reference	-
8–14 days	646 (50.4)	1.008 (0.726–1.399)	0.963
≥15 days	451 (35.2)	0.942 (0.668–1.328)	0.732
Doses of vaccine			
None	79 (6.2)	Reference	-
1	41 (3.2)	1.233 (0.564–2.691)	0.600
2	355 (27.7)	1.459 (0.876–2.430)	0.147
3	808 (63.0)	1.693 (1.042–2.750)	0.033

Abbreviation: SD, standard deviation; OR odds ratio; CI confidence interval.

**Table 2:** Psychological scores and relationship between variables in model.

	N (%)	Median (IQR)	I	II	III	IV	V	VI
I Stigma		29.0 (18.0)	1					
Low (score ≤ P_75_, 35)	978 (76.2)							
High (score > P_75_, 35)	305 (23.8)							
II Depression		40.0 (19.0)	0.109 ***	1				
No (score ≤ 42)	709 (55.3)							
Yes (score > 42)	574 (44.7)							
III Entrapment		1.0 (10.0)	0.487 ***	0.244 ***	1			
Low (score ≤ P_75_, 10)	965 (75.2)							
High (score > P_75_, 10)	318 (24.8)							
IV Decadence		2.0 (12.0)	0.522 ***	0.231 ***	0.785 ***	1		
Low (score ≤ P_75_, 12)	979 (76.3)							
High (score > P_75_, 12)	304 (23.7)							
V Education level	-	-	−0.059 *	−0.144 ***	0.156 ***	0.093 **	1	
VI Doses of vaccine	-	-	0.060 *	0.033	0.045	0.029	−0.076 **	1

Abbreviation: IQR, interquartile range (75th quartile minus 25th quartile); *: *p* value < 0.05; **: *p* value < 0.01; ***: *p* value < 0.001.

**Table 3:** Hierarchical regression analysis (*n* = 1283).

Model	Independent Variable	R^2^	β	t	*p*
Step 1		0.020			
	Education level		−2.884	−4.793	<0.001
	Doses of vaccine		0.448	1.352	0.177
Step 2		0.043			
	Education level		−2.695	−4.524	<0.001
	Doses of vaccine		0.377	1.147	0.251
	Stigma		0.148	5.551	<0.001
Step 3.1		0.160			
	Education level		−4.297	−7.523	<0.001
	Doses of vaccine		0.203	0.658	0.511
	Stigma		−0.038	−1.334	0.182
	Entrapment		0.360	13.340	<0.001
Step 3.2		0.147			
	Education level		−3.485	−6.151	<0.001
	Doses of vaccine		0.256	0.826	0.409
	Stigma		−0.040	−1.350	0.177
	Decadence		0.403	12.442	<0.001
Step 3.3		0.167			
	Education level		−4.126	−7.221	<0.001
	Doses of vaccine		0.207	0.673	0.501
	Stigma		−0.059	−2.016	0.044
	Entrapment		0.247	5.602	<0.001
	Decadence		0.171	3.252	0.001

**Table 4:** Results of path analysis (*n* = 1283).

Pathways	Point Estimate	Product of Coefficients			BOOTSTRAP 5000 TIMES 95% Cl			
					Bias Corrected		Percentile	
		S.E.	Est./S.E.	*p* Value	Lower	Upper	Lower	Upper
Total effect	0.145	0.028	5.162	<0.001	0.091	0.201	0.089	0.199
Direct effect								
Stigma→Depression	−0.059	0.031	−1.894	0.058	−0.121	0.004	−0.124	0.001
Indirect effect								
Stigma→Entrapment→Depression	0.125	0.022	5.669	<0.001	0.084	0.171	0.084	0.170
Stigma→Decadence→Depression	0.079	0.023	3.468	0.001	0.034	0.124	0.034	0.124
Total	0.204	0.019	10.912	<0.001	0.169	0.243	0.168	0.243
Entrapment vs. Decadence	0.046	0.041	1.140	0.254	−0.033	0.129	−0.033	0.128
Coefficient								
Stigma→Entrapment	0.507	0.034	15.123	<0.001	0.442	0.573	0.442	0.573
Entrapment→Depression	0.247	0.039	6.302	<0.001	0.173	0.325	0.171	0.324
Stigma→Decadence	0.461	0.027	16.981	<0.001	0.407	0.514	0.409	0.516
Decadence→Depression	0.171	0.048	3.576	<0.001	0.074	0.263	0.075	0.264
Stigma→Depression	−0.059	0.031	−1.894	0.058	−0.121	0.004	−0.124	0.001
Education level→Depression	−4.126	0.559	−7.380	<0.001	−5.248	−3.053	−5.254	−3.054
Doses of vaccine→Depression	0.206	0.302	0.683	0.495	−0.376	0.802	−0.389	0.789
Age moderated mediation effect								
Stigma→Entrapment→Depression								
Group 1 (18-39 years)	0.182	0.037	4.962	<0.001	0.115	0.126	-	-
Group 2 (40-71 years)	0.066	0.024	2.754	0.006	0.022	0.114	-	-
Group 1 VS Group 2	0.116	0.044	2.650	0.008	0.034	0.209	-	-
Stigma→Decadence→Depression								
Group 1 (18-39 years)	0.068	0.035	1.922	0.055	−0.002	0.135	-	-
Group 2 (40-71 years)	0.081	0.029	2.845	0.004	0.028	0.139	-	-
Group 1 vs. Group 2	−0.014	0.046	−0.303	0.762	−0.103	0.078	-	-

S.E. standard error; Est. estimate, CI confidence interval; *p* value < 0.05 was considered significant.

## Data Availability

The data are not publicly available due to privacy restrictions.

## Supplementary Materials

The following supporting information can be downloaded at: https://www.mdpi.com/article/10.3390/ijerph192013006/s1, Table S1. Factor loading of the Chinese version of the two subscale of Social Impact Scale (SIS) (*n* = 628); Table S2. Confirmatory factor analysis parameter estimation of the two subscale of SIS (*n* = 655); Table S3. Validity and reliability of the ES; Table S4. Confirmatory factor analysis parameter estimation of entrapment (*n* = 655); Table S5. Validity and reliability of the DS; Table S6. Confirmatory factor analysis parameter estimation of decadence (*n* = 655); Table S7. A multivariate logistic regression model of depression; Table S8. Logistic regression of depression with backward elimination/Change-In-Estimate procedure; Table S9. Parameter estimates for Mediation Analysis in the validation group (*n* = 298); Figure S1. The mediating role of entrapment and decadence in the relationship between stigma and depression in the training group (*n* = 985).
