# Supplementary material for: Social Stigma and Depression among Asymptomatic COVID-19 Carriers in Shanghai, China: The Mediating Role of Entrapment and Decadence
Source: Int J Environ Res Public Health. 2022 Oct 11;19(20):13006. doi: 10.3390/ijerph192013006 (PMC9602397; doi:10.3390/ijerph192013006)
Supplement: Supplementary file 1 [file ijerph-19-13006-s001.zip › ijerph-1897692-supplementary.pdf]

Supplement

I. Psychometrics

Participants were randomly divided into two groups based on a random number generator to be used to perform exploratory factor analysis (EFA; n = 628) and confirmatory factor analysis (CFA; n = 655), respectively, to evaluate the construct validity of the Social Impact Scale (SIS), Entrapment Scale (ES), and Defeat Scale (DS).

1. Social Impact Scale (SIS)

Construct validity

EFA was performed using data of the first group using IBM SPSS Statistics 26.0 (IBM Corp., Armonk, NY, USA). Principal component analysis with covariance matrix, eigenvalues greater than 1 and varimax rotation was used. Results of Kaiser-Meyer-Olkin (KMO) Test (KMO measure of sampling adequacy = 0.967) and Bartlett's Test of Sphericity (χ^2 = 14072.922, p < 0.001) indicated that the data were suitable for factor analysis. Four common factors were extracted, which accounted for 69.613% of the variance. Detailed information on factor loading of two subscales used in this study was shown in Supp Table 1.

Supp Table S1. Factor loading of the Chinese version of the two subscale of Social Impact Scale (SIS) (n = 628)

No	Item	Factor Loading
1	My employer / co-workers have discriminated against me because of my illness	0.530

2	Some people act as though I am less competent than usual	0.526	
3	I feel I have been treated with less respect than usual by others	0.616	
4	I feel others are concerned they could “catch” my illness through contact like a handshake or eating food I prepare.	0.640	
5	I feel others avoid me because of my illness	0.719	
6	Some family members have rejected me because of my illness	0.662	
7	I feel some friends have rejected me because of my illness	0.707	
8	I encounter embarrassing situations as a result of my illness.	0.652	
9	Due to my illness others seem to feel awkward and tense when they are around me	0.682	
10	I feel set apart from others who are well		0.616
11	I have a greater need than usual for reassurance that others care about me		0.418
12	I feel lonely more often than usual		0.551
13	Due to my illness, I have a sense of being unequal in my relationships with others		0.591

14	I feel less competent than I did before my illness	0.802
15	Due to my illness, I sometimes feel useless	0.817
16	The illness have affected my social relationships.	0.647

Data of the second group were used to perform a CFA using Mplus Version 8.3 (Muthen & Muthen, Los Angeles, CA, U.S.). χ^2 = 1469.127, DF =246, χ^2/DF = 5.972, RMSEA = 0.088, CFI =0.909, TLI =0.898, SRMR =0.056. These model fit indices of the four-dimensional model suggested that the construct of the questionnaire was acceptable. Detailed regression coefficients of each used item were shown in Supp Table 2.

Supp Table S2. Confirmatory factor analysis parameter estimation of the two subscale of SIS (n = 655)

No	Estimate	Standard error	z value
1	0.511	0.026	19.443*
2	0.559	0.025	22.389*
3	0.596	0.024	24.547*
4	0.697	0.030	23.555*
5	0.745	0.028	26.223*
6	0.618	0.025	24.480*
7	0.700	0.025	27.718*
8	0.711	0.028	25.399*
9	0.735	0.029	25.192*

10	0.575	0.025	23.127*
11	0.607	0.031	19.695*
12	0.643	0.024	27.066*
13	0.651	0.025	26.503*
14	0.651	0.024	27.625*
15	0.641	0.023	27.883*
16	0.707	0.026	26.740*

*$p < 0.001$

Reliability

Cronbach's α was calculated to evaluate the internal consistency reliability and was 0.967. Split-half reliability was determined by Spearman-Brown coefficient, which was 0.975. These two coefficients were calculated using IBM SPSS Statistics 26.0 (IBM Corp., Armonk, NY, USA).

2. Entrapment Scale (ES)

The analysis method is similar to that of SIS. Results are shown in Supp Table 3 and Supp Table S4.

Supp Table S3. Validity and reliability of the ES

Indices	Value
**Construct validity**	
Exploratory factor analysis	

KMO measure of sampling adequacy	0.969
Bartlett's Test of Sphericity	$\chi2 = 11681.917, p < 0.001$
total variance explained	73.489%
Factor loading of the Chinese version of the 16-item ES	0.785 – 0.904
Confirmatory factor analysis	
$\chi2/DF$	11.76
CFI	0.898
SRMR	0.040
**Reliability**	
Cronbach's α	0.973
Spearman-Brown coefficient	0.976

Supp Table S4. Confirmatory factor analysis parameter estimation of entrapment (n = 655)

No	Estimate	Standard error	z value
1	0.679	0.022	31.334*
2	0.843	0.012	70.529*
3	0.774	0.016	47.574*
4	0.883	0.009	95.603*
5	0.804	0.014	55.612 *
6	0.798	0.015	53.789*
7	0.819	0.013	60.741*

8	0.745	0.018	41.462*
9	0.804	0.014	55.797*
10	0.826	0.013	63.578*
11	0.829	0.013	64.685*
12	0.857	0.011	78.258*
13	0.884	0.009	95.675*
14	0.883	0.009	95.009*
15	0.826	0.013	63.312*
16	0.888	0.009	99.669*

*$p < 0.001$

3. Defeat Scale (DS)

The analysis method is similar to that of SIS. Results are shown in Supp Tables S5 and S6.

Supp Table S5. Validity and reliability of the DS

Indices	Value
**Construct validity**	
Exploratory factor analysis	
KMO measure of sampling adequacy	0.952
Bartlett's Test of Sphericity	$\chi 2$ = 8621.107, p < 0.001
total variance explained	70.969%
Factor loading of the Chinese version of the 16-item ES	

decadence (13 items)	0.778-0.891
low achievement (3 items)	0.744-0.892
Confirmatory factor analysis	
χ2/DF	5.690
CFI	0.946
SRMR	0.034
**Reliability**	
Cronbach's α	0.904
Spearman-Brown coefficient	0.923

Supp Table S6. Confirmatory factor analysis parameter estimation of decadence (n = 655)

No	Estimate	Standard error	z value
1	0.724	0.019	37.620*
3	0.764	0.017	45.115*
5	0.801	0.015	54.374*
6	0.844	0.012	70.521*
7	0.864	0.011	80.805 *
8	0.776	0.016	47.733*
10	0.736	0.019	39.648*
11	0.838	0.012	67.517*
12	0.874	0.010	87.391*

13	0.878	0.010	90.020*
14	0.864	0.011	81.062*
15	0.873	0.010	86.698*
16	0.852	0.011	74.271*

*$p < 0.001$

II. Covariate Selection

Supp Table S7. A multivariate logistic regression model of depression

variables	β	SE	Wals	P	OR
Age	-.003	0.006	0.292	0.589	0.997
Sex	0.018	0.119	0.024	0.878	1.018
Education level	-0.568	0.136	17.420	<0.001	0.567
Marriage status	-0.076	0.145	0.277	0.599	0.926
Length of time from diagnosis	-0.050	0.085	0.346	0.556	0.951
Doses of COVID-19 vaccines	0.145	0.072	4.128	0.042	1.157
Stigma	0.012	0.006	4.551	0.033	1.012

Supp Table S8. Logistic regression of depression with backward elimination / Change-In-Estimate procedure

variables	β	SE	Wals	P	OR
**Step 1 removal of age**					
Sex	0.015	0.118	0.017	0.896	1.016
Education level	-0.555	0.134	17.176	<0.001	0.574
Marriage status	-0.115	0.126	0.836	0.361	0.891
Length of time from diagnosis	-0.051	0.085	0.361	0.548	0.950
Doses of COVID-19 vaccines	0.144	0.071	4.035	0.045	1.154
Stigma	0.012	0.006	4.538	0.033	1.012
**Step 2 removal of sex**					
Age	-0.003	0.006	0.285	0.593	0.997
Education level	-0.564	0.134	17.823	<0.001	0.569
Marriage status	-0.075	0.145	0.266	0.606	0.928
Length of time from diagnosis	-0.050	0.085	0.342	0.559	0.951
Doses of COVID-19 vaccines	0.145	0.072	4.123	0.042	1.156
Stigma	0.012	0.006	4.594	0.032	1.012
**Step 3 removal of marriage status**					

Age	-0.005	0.005	0.850	0.357	0.995
Sex	0.013	0.118	0.012	0.913	1.013
Education level	-0.559	0.135	17.173	<0.001	0.572
Length of time from diagnosis	-0.051	0.085	0.364	0.546	0.950
Doses of COVID-19 vaccines	0.142	0.071	3.967	0.046	1.152
Stigma	0.012	0.006	4.434	0.035	1.012
**Step 4 removal of length of time from diagnosis**					
Age	-0.003	0.006	0.307	0.580	0.997
Sex	0.017	0.118	0.020	0.888	1.017
Education level	-0.562	0.136	17.177	<0.001	0.570
Marriage status	-0.079	0.145	0.295	0.587	0.924
Doses of COVID-19 vaccines	0.148	0.071	4.265	0.039	1.159
Stigma	0.012	0.006	4.668	0.031	1.012

Note: The change in OR of stigma in each step is calculated with respect to the OR=1.012 in the initial model (Supp Table 7)

III. Temporal Validation

Data were randomized to training group and validation group in a 3:1 ratio. Then the bias-corrected percentile bootstrapping method was used for temporal validation.

Supp Figure S1 The mediating role of entrapment and decadence in the relationship between stigma and depression in the training group (n = 985).

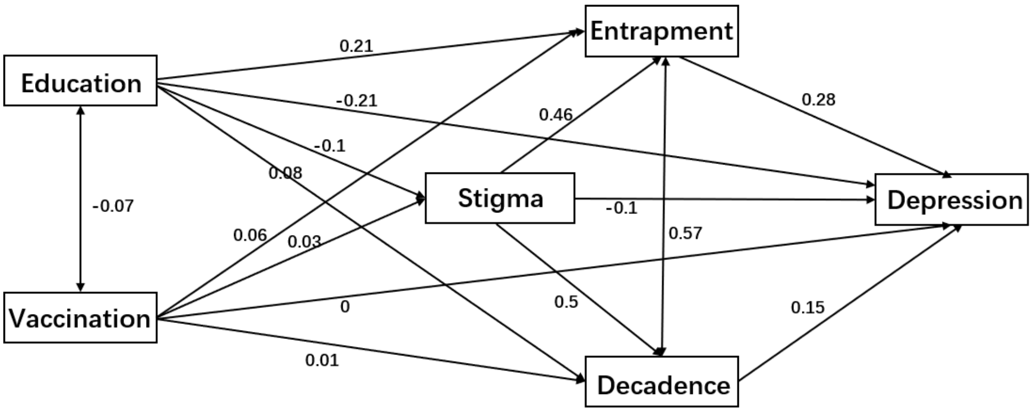

Supp Table S9. Parameter estimates for Mediation Analysis in the validation group (n = 298)

	Point Estimate	Product of Coefficients			95% Confidence Interval	
		**S.E.**	Est./S.E.	P value	Lower	Upper
**Direct effects**						
Stigma→ Depression	-0.101	0.048	-2.074	0.038	-0.203	0.005
**Indirect effects**						
Stigma→ Entrapment→ Depression	0.128	0.036	3.594	< 0.001	0.069	0.197
Stigma→ Decadence→ Depression	0.076	0.037	2.043	0.041	0.008	0.141
**Total effects**						
Stigma→ Depression	0.104	0.045	2.309	0.021	0.004	0.188
**Total indirect effects**						
Stigma→ Depression	0.204	0.029	6.951	< 0.001	0.149	0.259
**Residual covariances**						
Entrapment ↔ Decadence	0.574	0.043	13.360	< 0.001	0.446	0.722

Note: education level, doses of vaccine were controlled as covariates in the path analysis
